# Dynamics between earthquakes, volcanic eruptions, and geothermal energy exploitation in Japan

**DOI:** 10.1038/s41598-023-31627-3

**Published:** 2023-03-21

**Authors:** Thanushika Gunatilake

**Affiliations:** 1 Center for Hydrogeology and Geothermics (CHYN), University of Neuchâtel, Neuchâtel, 2000 Switzerland; 2 Present Address: ETH Zürich, Zürich, Switzerland

**Keywords:** Geodynamics, Geology, Geophysics, Seismology, Tectonics, Volcanology

## Abstract

Intruding magma brings high temperatures close to the surface, thus offering possibilities for harnessing large amounts of heat for geothermal exploitation. Mount Aso in southern Japan showed frequent volcanic activity during 2016, accompanied by significant earthquake activities with tens of thousands of aftershocks (Kumamoto sequence). Here we investigate the influence of earthquake/volcanic activity on the future productivity of nearby geothermal power plants to determine whether the activity is detrimental or beneficial to energy exploitation. Model results show an increase in ${\text {CO}}_2$ pressure and temperature with a spatio-temporal correlation between modeled earthquake locations and aftershock decay rates along the entire sequence, showing that seismic activity opened pre-existing vertical cracks providing pathways for the ascending magma. Interestingly, the minor but still significant eruption of Mount Aso in October 2021 may have enhanced future geothermal power generation, indicating a vigorous and active system, possibly increasing the future geothermal power production.

## Introduction

**Figure 1:**
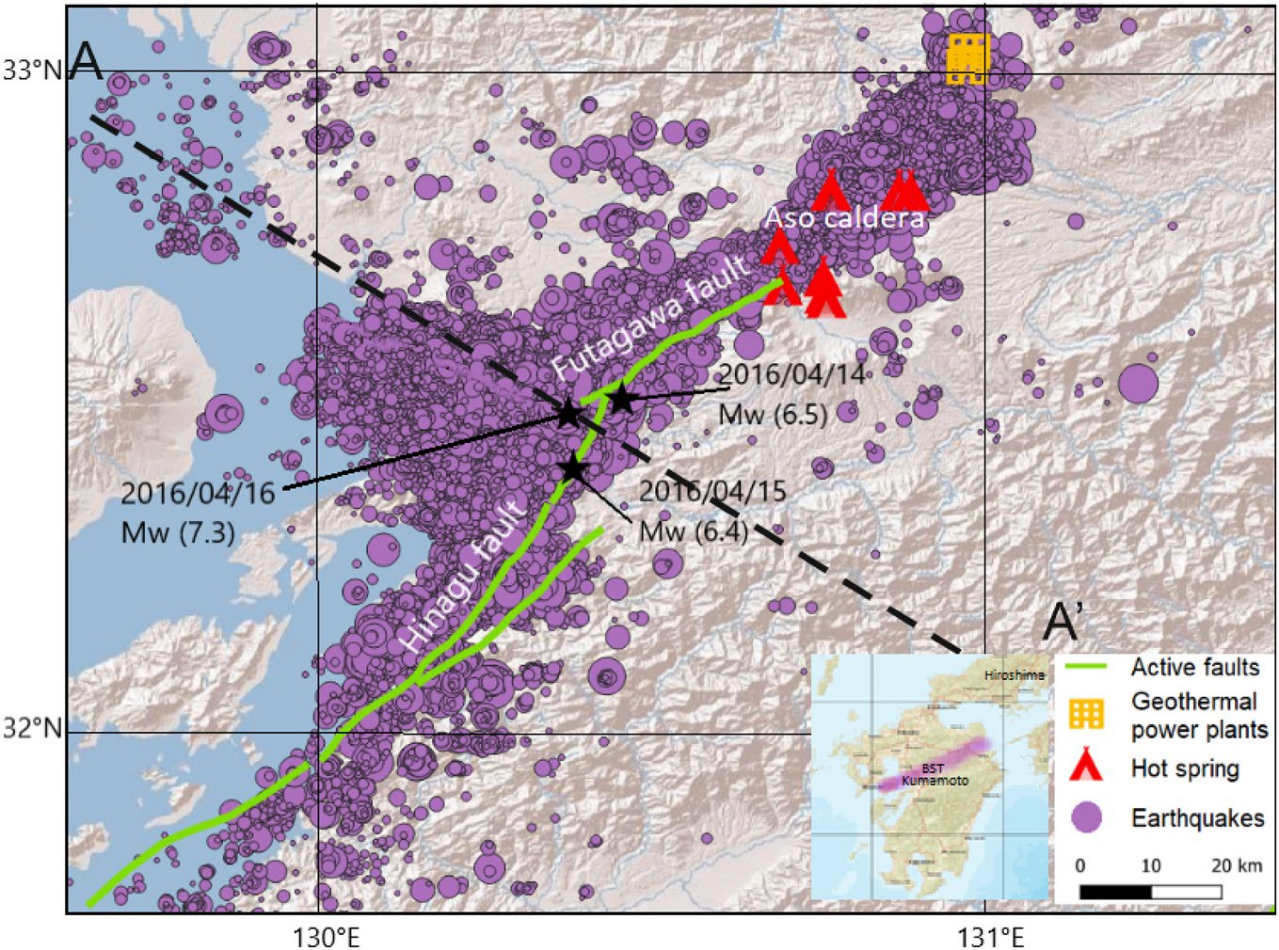
Digital elevation model of the study area, including hypocenters (purple dots) of 50.406 aftershocks from the Kumamoto earthquake sequence 2016 in southern Japan. Circle sizes refers to the earthquake magnitude, the black stars show the three main earthquakes, the green line shows the fault direction, and the red symbols show the hot volcanic springs of the Aso caldera and yellow boxed indicate the geothermal power plants (Hatchōbaru and Otake). The Beppu–Shimabara Trench (BST) is shown in purple in the subfigure. Figure generated with QGIS 3.16.15 Geographic Information System, http://qgis.osgeo.org.

Geothermal power plants close to active volcanic structures benefit from the elevated geothermal gradients^1^. However, volcanic activity is often associated with fluid triggered earthquakes with numerous aftershocks^2–7^. Japan is situated on the Pacific Ring of Fire and is known for its active volcanoes in a complex tectonic environment, in which the Pacific and Philippine plates are subducting beneath the Okhotsk and Eurasia plates at rates of 8–9 cm/year and 5–6 cm/year, respectively^8–10^. Over the years, destructive earthquakes have been recorded along the subduction zone as well as shallow crustal earthquakes away from plate boundaries as a result of internal deformation of tectonic plates. Figure 1 shows the well-located 2016 Kumamoto earthquake-aftershock sequence on Kyushu Island in southwestern Japan compiled by the Japan Meteorological Agency (JMA). This sequence started on April 14 with a $M_w$ 6.2 event at a hypocentral depth of 9 km, followed by another major earthquake ($M_w$ 6.0) on April 15 along the northern part of the Hinagu fault at a depth of 8 km^11,12^. On April 16, 2016, another earthquake ($M_w$ 7.0 ) at a depth of 10 km was recorded along the active Futagawa Fault and designated the mainshock of this sequence^13,14^.

Simultaneously, volcanic activity was recorded in the Aso Caldera, one of the largest active volcanoes in the world and with a cross-sectional area of 380 km$^2$. This volcano is known for pyroclastic eruptions, including ash emissions, strombolian eruptions, and phreatomagmatic activity^15^. High volcanic tremor activity around Mount Aso was observed before the Kumamoto earthquake^16^, and the higher activity level continued after the earthquake. Exsolution of high pressure gasses from the magma opened preexisting new pathways for the rising viscous magma^17,18^. Volcanic eruptions then occur as a result of increased pressure in a shallow magma reservoir caused by upwelling fluids from a deep magma chamber^19^. The Aso volcano erupted about 7 h after the April 14th main shock and continued its activity until October 8, 2016, where a major eruption occurred and discharged 15,000 tons of ${\text {SO}}_2$^20,21^. The seismic activity decreased after the the October 8th volcanic eruption, indicating reduced pressure within the system caused by the discharge of mass. Interestingly, satellite and ground-based thermal observations detected a rise in subsurface temperature during the Kumamoto earthquake sequence in April 2016 and by the end of the volcanic eruption in October 2016, reaching values well above $300\,^{\circ} \,\hbox {C}$^22^. These changes are consistent with the long-term well temperature-depth profiles gathered in the study area^23^.

The Hatchōbaru geothermal power plant capitalises on the high geothermal gradients in southern Kyushu^24,25^ and has been in operation since 1977. It is the largest geothermal power plant in Japan with a capacity of 112 MW. The neighboring Otake Power Plant with a capacity of 12.5 MW has been in operation since 1967^26–28^. The geothermal reservoir is located at depths between 500 and 1500 m at fluid temperatures ranging from 290 to 300 $^\circ$C^29,30^.

Previous studies revealed the importance of fluid pressure on the entire 2016 Kumamoto earthquake aftershock sequence, based on co-seismic stress changes, inversion analysis of pore fluid pressure, with multiple geophysical investigation including resistivity structures and tomographic imaging^31–33^. The rise of magma towards the subsurface is related to the release of gases (water vapor (${\text {H}}_2$O), carbon dioxide (${\text {CO}}_2$), and sulfur dioxide (${\text {SO}}_2$)), which then continues to propagate towards the surface^34^. The increase in temperature in the system enhances chemical reactions such as thermal decomposition and imposes additional high-pressure sources. In addition, there is evidence of coseismic hydrochemical changes during the the Kumamoto earthquake of 2016 ($M_w$ 7.3) and increased the ${\text {CO}}_2$ concentration in the system^35^. In other studies, soil ${\text {CO}}_2$ flux at Aso Volcano was continuously monitored from January 2016 to November 2017 using an accumulation chamber system, indicating a higher magmatic ${\text {CO}}_2$ anomaly after the October 2016 volcanic eruption due to the magma migration and volcanic gas from depth^20^. The aim of this work is to numerically investigate the role of fluids on the Kumamoto earthquake sequence and the subsequent volcanic eruption on the geothermal power production in close vicinity of the Aso Caldera. The model, described in detail below, investigates hydraulic behavior using a 2D model of non-linear pressure diffusion with a source term coupled with an advection–diffusion thermal model. The Kumamoto earthquake sequence, hot springs of the Aso caldera and Geothermal Power plants are projected onto the A–A′ profile (Fig. 1), which is perpendicular to the fault direction.

## Geological setting and model setup

Aftershocks of the Kumamoto earthquake occurred between Kumamoto City and the Aso Caldera located in the Beppu–Shimabara Trench (BST), a NE–SW volcanic belt that runs across central Kyushu^15,36^. This graben is characterized by numerous east–west trending active faults and volcanic activity^26,37^. The Aso caldera has a diameter of 24 km in the north–south direction and 18 km in the east–west direction^38,39^. In particular, the central cone, the Nakadake volcano with a composition ranging from basalt to basaltic andesite^20^ is an active volcano that erupts frequently^16^. It showed an increased level of activity during the Kumamoto earthquake sequence, compared to previous and subsequent years^40^. A time history of volcanic activity and seismic events from 2013 to 2019 is shown in the Supplementary.

Figure 1 shows more than 50,000 seismic events ($M_w$ > 1) in the year following the April 14th, 2016 Kumamoto earthquake and the ENE-WSW-oriented Futagawa and SWS-NEN-oriented Hinagu faults that ruptured during the event. This right-lateral strike-slip fault system is one of the major active fault systems on Kyushu Island^36,37,41^. Finally, Fig. 1 presents volcanic hot springs of the Aso caldera and two geothermal power plants, located close to the caldera.

A conceptual model of the study area was derived from electrical resistivity inversions performed at the active crater of the Nakadake central cone of Aso volcano using audio frequency magnetotellurics (AMT)^42^. The crustal structure beneath the Aso caldera, is characterized by pre-Neogene basement rocks and Quaternary volcanics inferred from a low-resistivity (1–10 $\Omega$m) signal. Seismic inversion studies and tomographic data confirm this sequence^43,44^.

**Figure 2:**
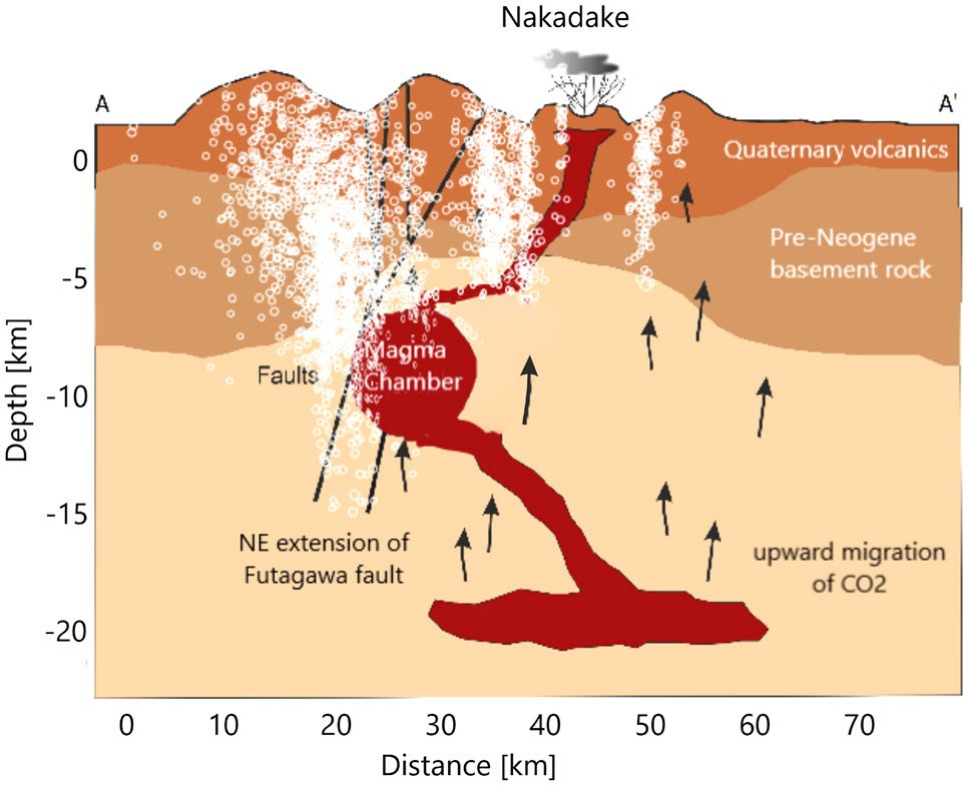
Conceptual model for the study area along profile A–A′ (Fig. 1) based on previous geophysical investigation. The aftershocks of the 2016 earthquake sequence with $M_w>$ 2.5 (white) is superposed to the conceptual model.

Further geophysical observations reveal a conductive zone that dips steeply to the north-northeast, which is highly fractured, and is filled with acidic hydrothermal fluids^42,45^. These fluids are transported from the low-lying main magma chamber into the Nakadake crater^46^. The core of the magma chamber is located at a depth of 5–6 km below the Aso caldera with an almost spherical shape of radius of 2 to 3 km and a volume of about 100 km$^3$^47,48^. However, this chamber cannot explain the massive volcanic eruption of ejecta, which covered more than 600 km$^3$ of space 90,000 years ago^49^. Recent studies of petrological analyses provide insight into the ongoing degassing process and revealed an over-pressurized magma chamber at a depth of 6 to 11km caused by gas accumulation^50,51^. Previous studies discovered another low-velocity layer at depths between 10 and 24 km below the southwestern part of the caldera^43,47^. Based on those geophysical studies, Fig. 2 shows a conceptual model of the study area, with the three different lithological units, the magma chamber, the fault system (to the southwest of the magma chamber) and micro fractures (to the northeast of the magma chamber). The position and the orientation of the faults is constrained by both the recorded 2016 Kumamoto earthquake-aftershock sequence and dip angles and slip distribution of the faults presented in other studies^33,52^. The geometry of the model is constrained by geophysical observations, especially through seismic and electrical resistivity studies^45,53^. Therefore, the environmental setting shown in Fig. 2 is used as the geological background model for the numerical simulations in this work.

Figure 2 also shows the 2016 Kumamoto seismic sequence superposed on the conceptual model and suggests a correspondence of earthquake locations with the identified fault structures on the southwestern side of the magma chamber. The seismicity on the northeastern side of the magma chamber agrees with the identified micro fractures. Thus, the conceptual model coincides with the dynamics of the earthquake propagation and formed the basis of this study.

## Results

**Figure 3:**
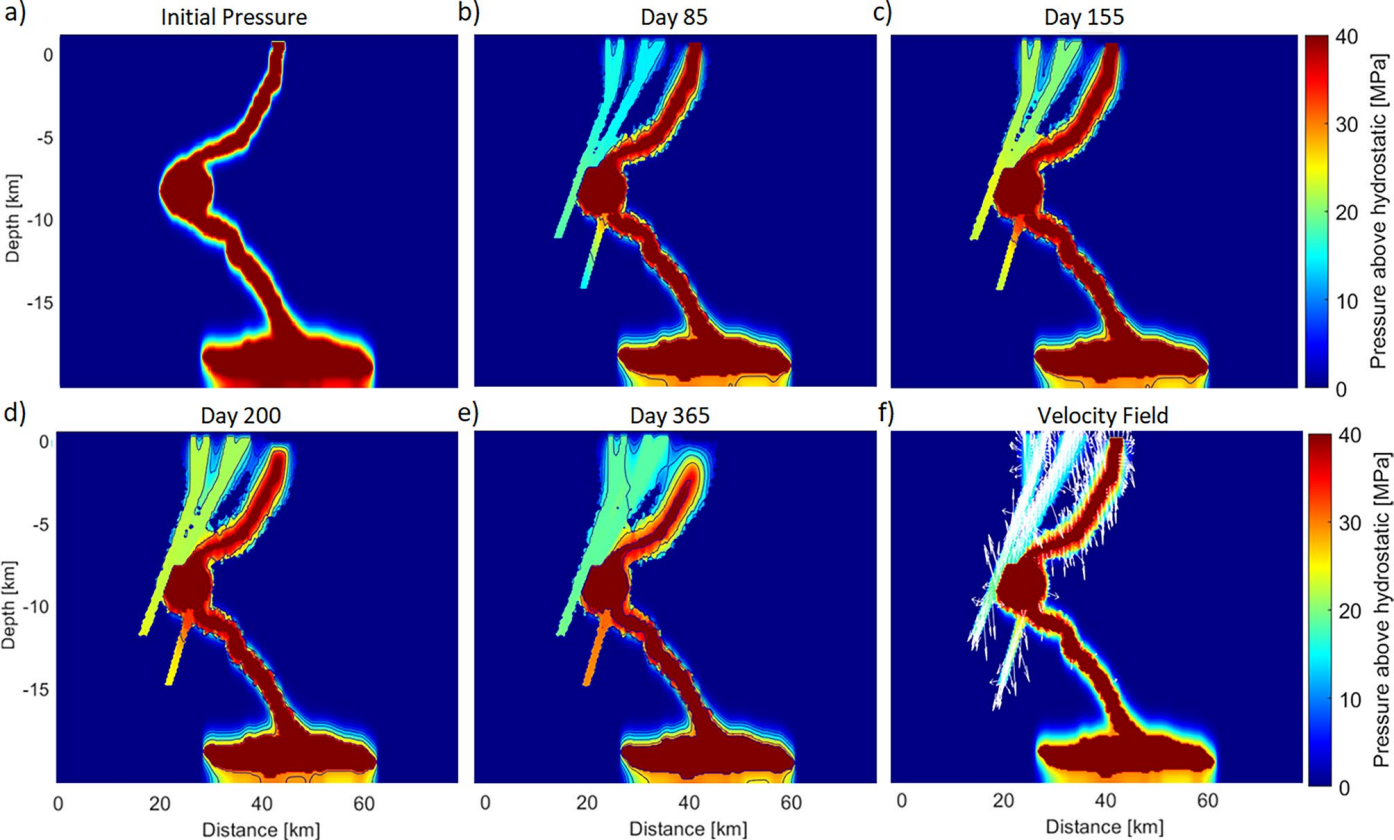
Calculated fluid pressure. (**a**) The steady state pressure distribution at the beginning of the simulation. (**b,c**) The fluid pressure is increasing within the faults toward the surface. (**d,e**) After the massive volcanic eruption on October 8, 2016, the fluid pressure decreases. The contour lines clearly illustrate these changes along the model. (**f**) The velocity field reflects the stream of the fluid pressure through the porous medium and show both the pressure propagation into the lower part of the chamber along the faults and into the upward oriented faults, allowing the rising of magmatic fluid towards the surface.

Simulations run for 365 days, which covers the duration of the aftershock sequence from April 2016 and volcanic eruption until volcanic silence in April 2017. Figure 3a shows the initial equilibrium pressure field at the start of the simulation. Figure 3b,c include the first phase of the Kumamoto earthquake sequence and the volcanic eruption on the 8th of October. Figure 3d,e show the calculated fluid pressure field from the end of the eruption until the overall cessation of significant seismicity. Pressure diffuses from the high-pressure magma chamber into the fault structures and tends to migrate both upwards and downwards along the faults. The simulation results of day 155 (reflecting the period immediately before the volcanic eruption on October 8, 2016) reveal an increase in pressure within the fault structures southwest of the caldera (Fig. 2), which triggers the subsequent seismicity. Figure 3d shows the decreased conduit pressure, consequently reducing the pressure in the faults, due to the breaking of the seal and the change in top boundary conditions mimicking the pressure release during the volcanic eruption. At the end of the simulation (Fig. 3e), a further decrease in the pressure gradient within the fault structure and conduit is observed, although an overpressure of about 20 MPa leads to further seismicity. Figure 3f shows the Darcy velocity vectors from the pressure field (Eq. 5) at each nodal point at 100 days. The flow vectors show both upward and downward flow and reflect the migration of fluid pressure along the conduit and its surrounding. The flow downwards is a result of a fault cutting the magma chamber and instigating a fluid pressure gradient towards the depth. Additionally, the fluid pressure migrates along the conduit and expands to its surrounding. The observed seismic activity in the northeastern and southwestern parts of the Nakedake volcano can be explained by the fluid-pressure increase along the fault structures and the conduit.

The temporal distribution of aftershock rates has been described by empirical laws, such as the Omori’s Law (later extended to the Omori–Utsu Law) in 1894. This law states that aftershock frequency decays as an inverse power law over time. Omori’s law is one of the fundamental empirical laws and is widely used to analyze seismic data. The modified Omori’s law describes the frequency of earthquakes after the main shock and states that the rate of aftershocks decreases hyperbolically with time:

1 $$\begin{aligned} N(t)=\frac{K}{(t+c)^{p}}, \end{aligned}$$

where *N* is the number of earthquakes as a function of time *t* following the mainshock, *K* is the aftershock productivity, and *c* and *p* are empirical constants. The *c* value relates describes the time lag until Omori behavior, but it difficult to measure because immediate aftershocks get drown in the seismic record. The exponent *p* determines how fast the aftershock rate decreases with time and often has a value close to 1.0^54,55^. Miller recently proposed that the p-value is a measure of tectonic stress state available to heal co-seismically generated permeable fracture networks.

**Figure 4:**
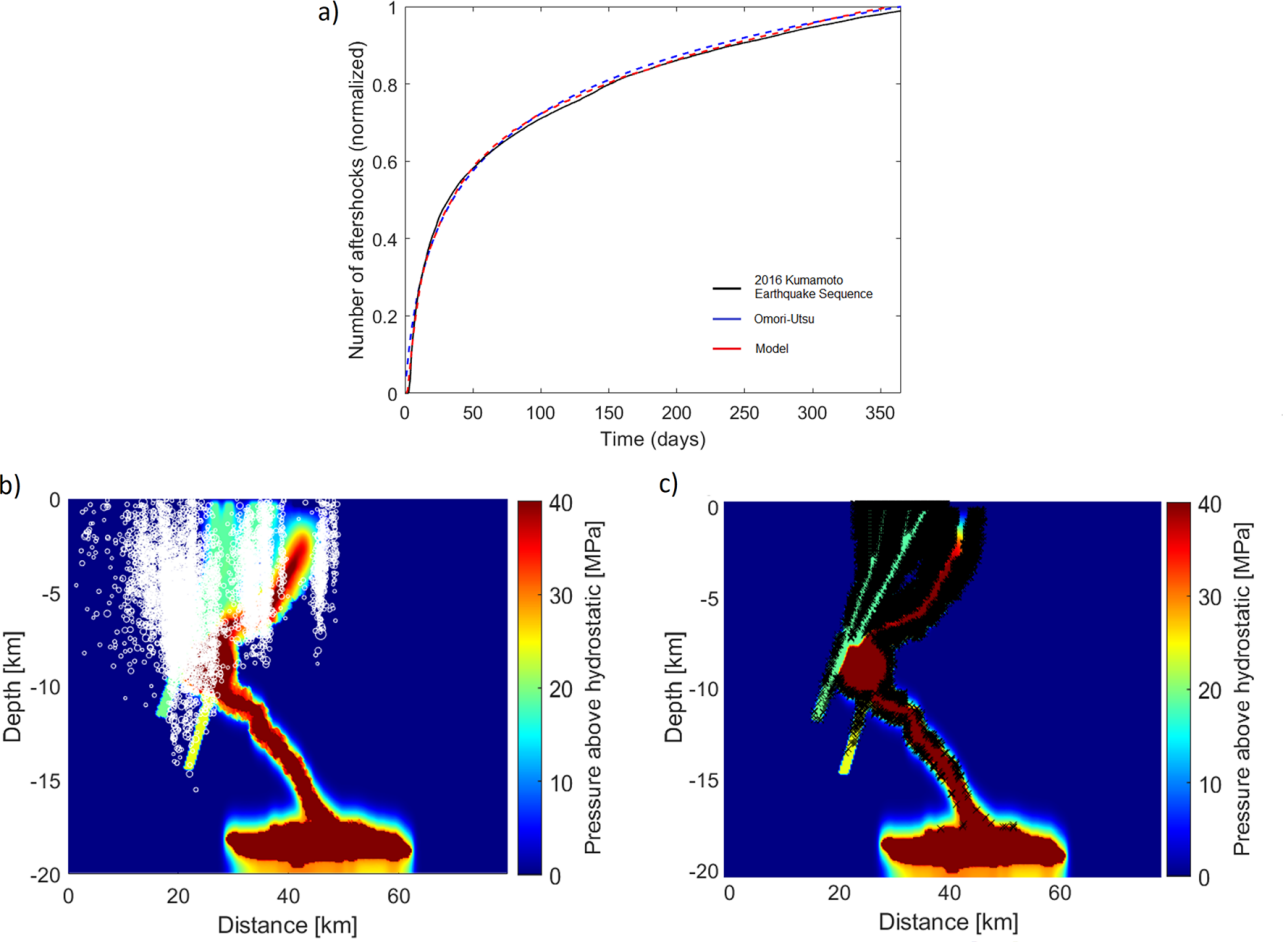
Temporal and spatial comparison. (**a**) Comparison of observed cumulative aftershocks for profile A–A′ with Omori–Utsu law and numerically triggered earthquakes using the model presented. The data constrains the value of $\alpha$, which in this case is $1/\alpha = 105$ days. (**b**) Spatial distribution of the recorded seismic data (white) (**c**) and numerically triggered hypocenters (black).

Figure 4a shows observed cumulative aftershocks (black) superposed with the Omori fit and the modeled cumulative aftershocks (red) for the profile A–A′ across the study area. Both attempt shows good agreement, while the Omori fit obtained by using the p-value 1.05, and the physical model proposed in this work obtained by varying the dominant parameter in the model, $\alpha$ that appears in both the permeability (Eq. 3) and source pressure (Eq. 4). When tectonic stresses shut down the permeability network created by the earthquake/aftershock, the healing process begins. Conceptually, $\alpha$ reflects the duration needed for the permeability to heal and the timescale of the source generation^56,57^. The value for $\alpha$ is constrained by the data and shows a recovery time $1/ \alpha = 105$ days. Figure 4b shows the calculated fluid pressure field (at 365 days) and recorded seismic data (white) and Figure 4c shows the numerically triggered earthquakes (black) superimposed to the calculated fluid pressure. The evolved fluid pressure field is controlled by the permeability dynamics within the model, which is controlled by $\alpha$. The fluid pressure increases within the fault structure and around the conduit and triggers earthquakes at the corresponding nodal points. The entire spread of fluid pressure, temperature, and fluid velocity field throughout the simulation is attached as a Videos 1, 2 in the Supplementary.

**Figure 5:**
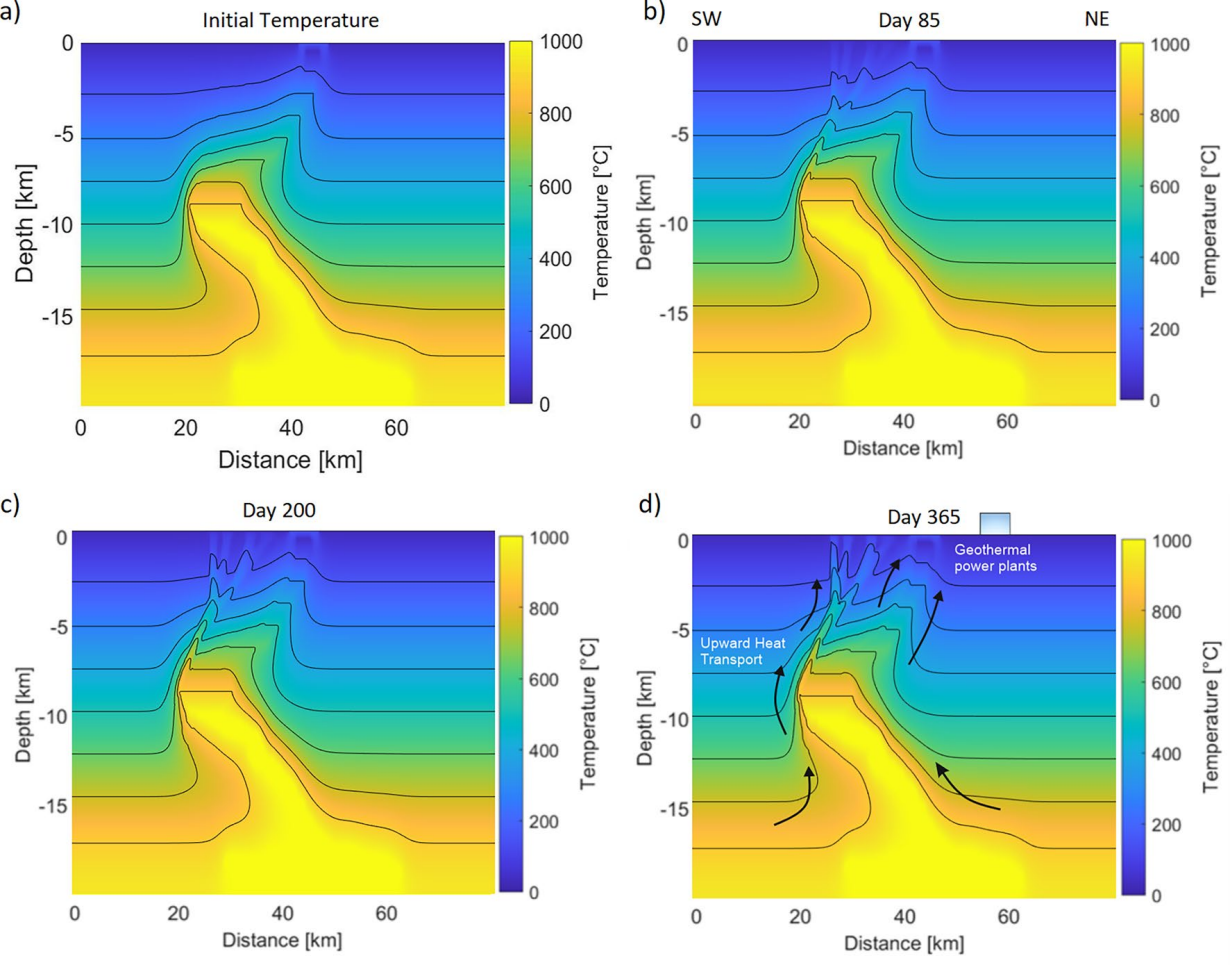
Advection–diffusion of temperature for different time steps. (**a**) Initial temperature distribution. (**b,c**) The heat migrate from the magma chamber to the faults and upwards within the conduit. (**d**) Temperature distribution at day 365 and location of geothermal power plants illustrating the upward migration of the heat. The entire temperature evolution is attached as a Videos 1, 2 in the Supplementary.

Figure 5a depicts the initial temperature field, which corresponds to 100 years of thermal conduction from a heated magma chamber. The figure exhibits a slight temperature rise in the predefined magma conduit, with a gradual increase in temperature within the conduit and fault system. Furthermore, the temperature distribution displays smooth gradients due to heat flux into the surrounding rock. At day 85 (Fig. 5b), heat is transported away from the chamber as it migrates upwards along the faults (Fig. 5b,c). These observations indicate an advection-dominant heat transport process occurring in the system. Figure 5d shows the temperature distribution at the end of the simulation (day 365), indicating the temperature near the surface around the volcano has increased to roughly 40 $^\circ$C. Ubiquitous heat transport to the northeast and southwest can be observed, which would directly impact geothermal exploitation potential. The direct temperature increase around the geothermal power plants is obviously limited. Note that microfractures in the northeast are not implemented, but this would create additional pathways for heat transport towards the geothermal power plants. The comparably small temperature increase outside of preferential pathways is in agreement with other thermo-hydro-mechanical models of natural seismicity^58^.

**Figure 6:**
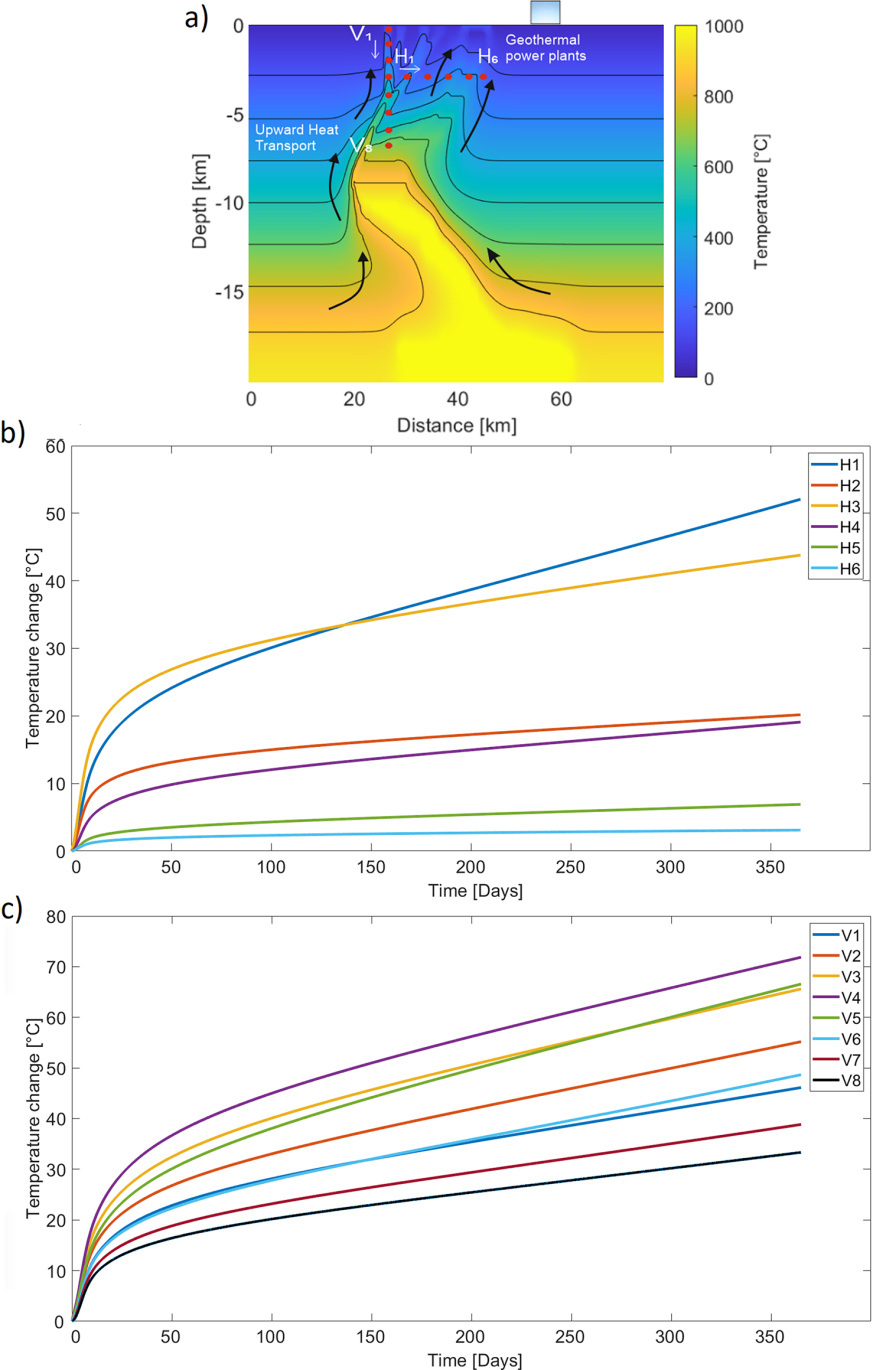
Temperature profiles. (**a**) Temperature distribution at 365 simulation day. The red dots indicate the location of the vertical and horizontal profiles. (**b**) Horizontally aligned temperature profiles (H1–H6) with a vast difference in temperature distribution. (**c**) Nine temperature distribution profiles show the temperature increase from top (V1) to bottom (V8).

An examination of vertical profiles (V1–V8) and horizontal profiles (H1–H6) quantifies the temperature changes at various observation points along the fault structure (Fig. 6a). Figure 6b shows the temperature variations for the horizontal locations, and much more diversity compared to the vertical locations. Locations H1 to H3, within the fault structure, show a rapid increase in temperatures over the simulated time span. H6 located in the vicinity of the geothermal power plants, shows a slight temperature increase (3 $^\circ$C). Figure 6c quantifies temperature profiles, aligned along the fault from bottom to top, and shows significant temperature increases due to advective heat flow. As expected, temperature cools towards the surface but all locations experience significant temperature increases in the southwestern part of the system. Figure 6c also shows a fairly rapid temperature increase that stabilizes over the simulated time. In general, a substantial temperature change was observed both horizontally (5–60 $^\circ$C) and vertically (25–75 $^\circ$C) at certain depths investigated. These results show that the active Aso volcano causes a step-like geothermal gradient and that there is the potential of harvesting the rising heat for for geothermal use. This model, which replicates thermo-hydro-mechanical processes, can be used to better understand, and potentially predict the temperature increase during an eruption and to determine whether it is detrimental or beneficial to energy exploitation.

## Discussion

This study focuses on understanding the relationship between seismic and volcanic dynamics in a single-phase flow, by coupling the non-linear diffusion problem with temperature advection and diffusion. I investigated the chemical and physical dynamics by considering permeability recovery and additional fluid sources via thermal decomposition, controlled by the alpha value. However, it should be noted that, in reality, the fluid in a magma chamber is likely to be a multiphase mixture, which will affect the diffusion and advection of fluids in the system. A single-phase fluid limit addressing the parameterizing fluid properties. While more advanced multi-phase models would be desirable, they cannot currently be combined with the necessary mechanical and dynamic-permeability models used in this work. Furthermore, the primary objective of the conceptual model is to achieve a comprehensive understanding of the geological characteristics of the study area by considering the dynamic interplay between volcanic and seismic activities in a physically and mathematically rigorous manner. However, to gain insights into the pressure diffusion of the system, it was assumed that the rocks in the system behave entirely in a brittle manner, without significant ductile deformation. In the future, it is crucial that research explores the impact of the brittle–ductile transition zone on volcanic and seismic activities, as there is a high likelihood that we currently underestimate the potential for deformation and strain within the volcanic region. However, investigating the effects of the transition zone on the mechanical properties of rocks under varying conditions of temperature, pressure, and deformation rates is a complex and challenging task, as it necessitates a rigorous scientific approach. Although, the outcomes of these investigations have the potential to advance the comprehension of geodynamical systems and enhance the ability to precisely model mechanical behavior. Understanding the underlying physical processes will ultimately enable us to make more dependable predictions regarding heat transport. Despite these limitations, the model provides valuable insights into the relationship between fluid flow and seismicity, and the results of the simulation are relevant for understanding the dynamics of a magma chamber. This work employed a coupled thermo-hydro-mechanical (THM) model to examine the pore pressure and temperature evolution in the year following the Kumamoto earthquake and dramatic aftershock sequence beginning April 15, 2016. A source term $Q_0 = 10^{-9}~{\text{s}^{-1}}$ is imposed on the activated faults and in the magma chamber to simulate the degassing process of active volcanic systems. The assumption of a source term in the model is a valid approach but it is important to note that further assumptions are needed to calculate the magnitude of the release in units of kg/s. These additional assumptions may include, for example, the density of the fluid being released and the area over which it is being released. Without these additional assumptions, it is impossible to accurately calculate the magnitude of the release in terms of kg/s. However, the source term in units of Pa/s still provides valuable information about the rate of pressure increase at the source, which can be used to understand the dynamics of the geothermal system.

The simulation of overpressure provides insight into the influence of fluid pressurization on the behavior of magmatic systems. However, neglecting effects of variable fluid density may lead to an overestimation of the fluid pressure in the upper part of the rock and an underestimation in the lower part, especially for cases with a significant vertical pressure gradient. On the other hand, neglecting variable temperature and pressure reduces the computational cost dramatically, and gives insights into a better understanding of the relationship between fluid pressure and the behavior of magmatic systems, which can improve our ability to identify indicators for imminent volcanic activity and respond to potential hazards. However, it is important to validate the results against observational or experimental data to assess the reliability of the simulation in future studies.

The findings of this work support a numerical model that proposes a conceptual understanding of how earthquake/aftershock sequences can breach hydraulic seals and form hydraulic connections between faults and magmatic chambers. This is in agreement with the quantitatively examined work of Nakagomi, 2021^31^ that demonstrated pore fluid pressures controlling the aftershock generation. The subsequent migration of high pore pressures triggered aftershocks primarily within and around the fault system and instigated advective heat transport. The model reproduces the temporal and spatial aftershock behavior of the earthquake catalog and shows good agreement with the recorded data. To capture the temporal and spatial evolution of the 2016 Kumamato earthquake sequence, I adjusted $\alpha _j$ until a suitable fit was obtained for the entire data set, as $\alpha _j$ directly influences the occurrence of aftershocks in the model. Thus, the model accurately reproduced observations with 1/$\alpha$ = 105 days, roughly corresponding to the inflexion point of the temporal aftershock occurrence (Fig. 4), which is then followed by a significant reduction in the rate of aftershocks. Furthermore, the 1/$\alpha$ value reflects the timescales of thermal decomposition and permeability recovery, which temporally corresponds to the shortly-followed volcanic eruption and the decrease of high-pressure fluids in the faults and conduit. From a geodynamic perspective, this might be related to the inflation of the volcanic magma detected at the surface roughly after 105 days (July 2016)^59^ .

Simulating advective heat transport in geothermal systems is a major challenge due to the unknown heterogeneity of the subsurface. The subsurface structure, rock type, and physical properties can greatly impact temperature distribution and heat transport, but these are often difficult to determine. This leads to significant uncertainties in the model predictions. Neglecting processes such as viscosity and density changes is a reasonable choice, as including them would result in a more complicated multiphase problem and pose computational difficulties, hindering the accuracy of the predictions and the determination of heat transport in geothermal systems. The simplified model used in the study is sufficient for understanding the seismic and heat behavior of the system, but the predictions may not be highly accurate due to simplifications made. Nevertheless, simulating the complex interactions between fluid flow, magma migration, and rock failure at once can improve the model accuracy and provide more accurate predictions of the temperature changes at depth in future studies. While the specific temperature distribution at a greater depth around the magmatic system is unknown, principal findings can be derived by comparing various initial temperature distributions. This study presented the initial temperature field after 100 years of conductive heating. Figure 6 in the Supplementary Materials assumes 200 years of thermal conduction, and show, that a smoother geothermal gradient between the magmatic system and the surrounding host system results in more pronounced temperature changes in the faults and in the vicinity of the surface. However, as the system is advection dominated, the temperature change in the growing distance from the magma chamber also depends strongly on the permeability of the system. Therefore, the influence of the subsurface temperature increase around the magma chamber is primarily local. Still, increased temperatures in the faults at greater depth might cause thermally triggered earthquakes in addition to pore-pressure driven earthquakes.

In the results, the heat transport is dominated by advection inside of the faults and volcanic structures, with comparably small temperature changes (via diffusion) in the vicinity of the fluid pathways. Determination of advective heat transport is usually challenging, as temperature measurements at greater depth are rarely available. Therefore, constraining the advective heat flow using earthquake propagation allows the reduction of uncertainties with respect to heat transport by providing a solid estimate of the flow velocity. The Miyakoshi 2020 study^23^ measured temperature changes in shallow wells in the study area and focused on the top 100 m of depth and found decreasing temperatures in some wells after the earthquake sequence, indicating increased fluid flow downwards. This supports the findings, as we observed numerous earthquakes at shallower depths and a small temperature decrease (V8 profile). In this model, each numerical seismic event causes permeability recalculation with each time step, resulting in enhanced fluid flow and confirms the effect of earthquakes on permeability and thermal structure. Despite the decrease in shallow temperatures, we still see an increase in temperatures at depths relevant for geothermal power plants, reinforcing the model approach presented. Based on the simulation results, it can be inferred that the volcanic and seismic activity in 2016 had a certain impact on the water temperature in the vicinity of the geothermal power plants. This effect may have contributed to an increase in the potential for geothermal energy. However, the major earthquake destroyed power lines and the power plant had to be shut down for some time. The recent volcanic eruption in October 2021 possibly influence the geothermal energy potential. Interestingly, this eruption was unaccompanied by concomitant seismic activity, suggesting that the high fluid pressure needed to drive seismic sequences remained subdued. Additional studies are clearly warranted to investigate the October 2021 eruption and its influence on geothermal power production.

## Conclusion

The April 2016 Kumamoto earthquake and subsequent aftershocks directly influenced volcanic activity at the Aso caldera. Observations and modeling suggest that the seismicity is directly related to pressure variations associated with the magmatic activity of the volcano. It can be speculated that the rupture of the mainshock initiated from the deep portion of a northwest-dipping fault plane along the Hinagu fault, which then triggered the Futagawa fault, propagated northeastward and upward, opening fluid pathways along the way. Entrained high-pressure fluids likely drove the aftershock sequence, and the subsequent fluid pressure dissipation allowed the expansion of the magmatic chamber and the eventual eruption of the Aso volcano. The heat around the geothermal power plant increased to some degree and suggests that the 2021 Aso eruptions are beneficial for geothermal energy potential and power generation. This research sheds light on the connection between fluid flow and seismic activity in a magma chamber and offers an opportunity for further study using a more comprehensive approach. Further research should be conducted to improve the accuracy of the model, taking into account the effects of different compositions of magma and fluid, crystallization and solidification processes, multiphase fluid flow, and the deformation of the magma chamber that may impact the heat transport in favor of geothermal energy.

## Methods: mathematical and numerical model

Over the previous decades, numerous approaches have been attempted to understand the cycle of earthquake and aftershock sequences. The most common approach is the stress transfer in earthquake occurrence due to changes in Coulomb failure stress ($\Delta$CFS)^60–62^. This technique was further advanced to include rate-state friction^63,64^. Statistical models such as ETAS (Epidemic Type Aftershock Sequence) assume that aftershocks can generate their own aftershock sequences. This model yields earthquake production rate comparable with the recorded seismic events^65,66^. However, this empirical magnitude-dependent aftershock productivity model currently lacks any physical basis. An alternative view for understanding earthquake propagation assumes that aftershocks are driven by the diffusion of fluids due to high pore pressure. The importance of fluids in tectonics and earthquake processes has increasingly been recognized over the past years^2,67–69^.

The mathematical model is based on diffusion of fluid pressure in a porous medium and is governed by:

2 $$\begin{aligned} \frac{dP}{dt} = \frac{1}{\phi \beta } \nabla \cdot \Biggl [\frac{k}{\eta } \nabla P \Biggr ]+ \frac{S}{\phi \beta }, \end{aligned}$$

where *P* is fluid pressure [Pa] above hydrostatic, *t* is time [s], $\phi$ is porosity [–], $\beta$ is the lumped compressibility of pore space and fluid [Pa$^{-1}$], *k* is permeability [m$^2$], $\eta$ is fluid viscosity [Pa s], *S* is a source term [s$^{-1}$]. Pore pressure diffusion is primarily responsible for the buildup of fluid pressure, which triggers seismic activity that promotes fracture opening. Subsequently, permeability increases^70,71^, which enhance further activation of the fault and opening of fractures, allowing the propagation of the high-pressure fluid. Thus, the post-seismic dynamics of permeability control the earthquake-aftershock decay rates. Over time, the tectonic relaxation and chemical precipitates shut down the fracture networks created by the earthquake/aftershocks resulting in the healing of the permeability networks. Therefore, I adopt the permeability model^56^, which includes a permeability recovery over a time scale initiated at the onset of a numerical earthquake.

3 $$\begin{aligned} k = kc_0 \cdot \exp(- \alpha t) \cdot exp(-\sigma _e / \sigma ^*), \end{aligned}$$

where $kc_o$ is a baseline permeability, $\alpha$ [s$^-1$] is the parameter controlling the permeability recovery for the duration *t* [s] and $\sigma ^*$ [Pa] constrains the permeability response to the effective normal stress $\sigma _e$ [Pa].

The decarbonization and dehydration process release large amounts of CO2 and water, which are accounted for in our model as an additional source term *S* and are mathematically expressed as:

4 $$\begin{aligned} S = Q_0e^{- \zeta t}, \end{aligned}$$

where $Q_0$ [s$^{-1}$] is introduced at regions of recent earthquakes and $\zeta$ determines the timescale *t* of this internal source generation. The time scales of thermal decomposition and permeability are treated in a similar way, and thus I assume $\alpha _j = \zeta _j$ for both permeability and source terms to minimize the number of free parameters. A more detailed description can be found elsewhere^3,56^.

Previous studies based on laboratory experiments show that thermal pressurization lubricates frictional interfaces associated with devolitization and highly generates overpressurized fluids^72^. Numerical thermo-hydro-mechanical–chemical (THMC) models investigate the reaction between a rising melt and a viscous solid coupled with heat transfer. The results indicate a dramatic reduction of effective viscosity upon shear heating, leading to thermal runaway and proving the presence of high-pressure fluid at depth^73^. Furthermore, there is numerical evidence that high-pressure fluids coincide with the degassing of ${\text{CO}}_2$ from the Earth’s mantle as a trigger and driver, for example, the entire 2008 Bohemian earthquake swarm^58,74^. This study explores the potential influence of the evolving fluid pressure and the resulting velocity on the temperature distribution. The Darcy velocity *q* [m/s] can be derived from the pore pressure model:

5 $$\begin{aligned} \vec {q} = \frac{-k}{\eta } \nabla P, \end{aligned}$$

which is used to calculate the advective heat transport term in the diffusion advection equation:

6 $$\begin{aligned} \rho c_p \frac{dT}{dt} = \lambda \nabla ^2 T - \phi (\rho c_p)_f \vec {v} \cdot \nabla \vec {T}, \end{aligned}$$

where $c_p$ is specific heat capacity [J/kg/$^\circ$C], *T* is temperature [$^\circ$C], *v* is pore fluid velocity ($v = q/\phi$) and $\lambda$ is thermal conductivity [W/m/$^\circ$C] of the fluid-solid system. Local thermal equilibrium is assumed. Thus, specific heat capacity and density on the right-hand side of the equation are system parameters, so fluid and rock properties are weighted by porosity, similar to thermal conductivity. After the weighting parameter variation for the different rock types is negligible, therefore, thermal conductivity is assumed homogeneous. The advection term on the right-hand side only incorporates fluid parameters as only the fluid is flowing relative to the porous matrix^75^.

To calculate the mechanics, I consider a hydrostatic pressure distribution:

7 $$\begin{aligned} P_f = \rho \times g \times h, \end{aligned}$$

where $\rho$ is density of the fluid, g is gravity and h depth of the fluid column. Obtaining the total pressure due to the weight of the fluid the effective normal stress on incipient slip planes is calculated as:

8 $$\begin{aligned} \sigma _n = \frac{\sigma _1 + \sigma _3 - 2P_f}{2} - \frac{\sigma _1 - \sigma _3}{2} \cos (2\theta ), \end{aligned}$$

where $\sigma _1$ and $\sigma _3$ are the maximum and minimum principal stresses [Pa], $\theta$ is the randomly distributed angle relative to the orientation oft he maximum principal stress, and $P_f$ is the fluid pressure above hydrostatic. To reflect the extended environment in the study area, the maximum $\sigma _1$ and minimum $\sigma _3$ principal stresses are horizontal and orthogonal, so $\sigma _3$ is assumed to be 0.62 $\cdot \sigma _1$^2^. In this configuration, the optimum failure plane is oriented about 60 degrees from $\sigma _1$ and a random distribution of incipient slip planes relative to principal stress directions is scattered throughout the numerical domain.

The shear stress $\tau$ on the virtual planes are:

9 $$\begin{aligned} \tau = \frac{\sigma _1 - \sigma _3}{2} \sin (2\theta ).\end{aligned}$$

A numerical aftershock is defined as a nodal grid point reaching the Mohr–Coulomb failure condition:

10 $$\begin{aligned} \tau \ge \sigma _n \mu, \end{aligned}$$

where $\mu$ is the friction coefficient [–].

The time and location of a each triggered numerical aftershock is recorded, and only one event is permitted for each computational node.

Due to the dependency, the permeability (K) in the domain is then calculated based on the normal stress.

11 $$\begin{aligned} K = k \times \exp(-\sigma _n/\sigma *). \end{aligned}$$

This calculation allows a stronger downward decrease in the normal stress, hence a heterogenous permeability which results in an asymmetric pressure diffusion with accelerated flow upwards and inhibited flow below the magma chamber^76^. The depth-dependence of permeability (Fig. 2b in Supplementary) is observed in crystalline rock in tectonically active areas and can be constrained in the presented model by considering the relationship between fluid flow and seismicity.

The numerical model represents the magma chamber, faults, and the surrounding rock with their characteristics for a 2D simulation. The material properties for different regions of the simulation are specified in terms of initial permeability and porosity. A 40 MPa overpressure above hydrostatic is assumed in the magma chamber and conduit, while hydrostatic pressure is assumed in the host rock neglecting the pressure and temperature dependence on fluid viscosity and density. I then examine the impact of overpressure on the dynamics of advective heat transport and effective normal stress.

Equations (2) (pressure diffusion) and (6) (Heat advection–diffusion) are solved numerically using implicit finite differences on a regular grid of 300 $\times$ 300 nodal points. The length of the domain is 80 km and the height of the domain is 20 km. The distance between grid points is the ratio between the number of grid points in each direction and the length and height respectively. The simulations run for 1 year with a time step of 0.1 days. No-flow boundary conditions are imposed on the side and bottom boundaries for the hydraulic model, and a constant hydrostatic pressure at the surface. The model includes a temperature-dependent viscosity for supercritical ${\text {CO}}_2$ with a value of $\eta = 1e^{-4}$ Pa s at the surface, resulting in $\eta = 1e^{-5}$ Pa s at the depth of the fault system. Following^77^ the pore space and fluid compressibility can be summarized as a lumped compressibility. The supercritical ${\text {CO}}_2$ has the flow properties of water because it is approximately 10 times as compressible but also one-tenth as viscous and is therefore assumed with a value of $\beta = 4e^{-9}$ Pa$^{-1}$^2^. Note that in this study the supercritical ${\text {CO}}_2$ is consider as a single-phase flow, which can be extended to a multi-phase flow considering ${\text {CO}}_2$, ${\text {H}}_2$O, and ${\text {SO}}_2$ in future work.

${\text {CO}}_2$ degassing in magma controls the eruptive power of volcanic systems due to rapid decompression of the rising magma. The magma ascending in a conduit is decompressed and volatiles exsolve as bubbles in the magma. As the bubbles in the magma grow during decompression, an interconnected bubble network forms through which permeable degassing can occur^78^.

Laboratory experiments demonstrate that magma with entrapped fluids (${\text {H}}_2$O and ${\text {CO}}_2$) are of 0.1–10 wt% and there porosity is greater than about 60 vol%^79,80^. Consequently, a porosity of 0.6 for the magmatic fluid is assumed and a porosity of 0.03 is assumed for the rock matrix^81^. Finally, $\sigma ^*$ is assumed to be 35 MPa^56^, permeability is constrained by laboratory experiments on ejected material and showed a permeability of $2.5e^{-13}$ m$^2$^82^, whereas the permeability of the fault is assumed to be higher ($1e^{-9}$ m$^2$) than of the host rock. The permeability of both the magma chamber and surrounding rock can be important factors in the dynamics of volcanic systems. In this study, I assume the permeability of the chamber to be higher than the surrounding rock at 5–10 km depths. Although, the permeability of a magma chamber can vary depending on its physical characteristics, and it is not always necessarily higher than that of the surrounding rock. The permeability of a magma chamber depends on various factors, such as the porosity of the magma and the surrounding rock, the temperature and pressure conditions, and the mineral composition of the rocks. For example, the permeability of a magma chamber can increase with temperature due to the reduction of viscosity of the magma, and it can also increase with pressure due to the compaction of the surrounding rocks. Additionally, the presence of fractures or faults in the rocks can significantly influence the permeability of the magma chamber. Figure 5a in the Supplementary File illustrates the permeability of the magma chamber to be lower than that of the surrounding rock. The numerical simulation presented in this study was conducted with the assumption that the permeability of the magma chamber is lower than that of the surrounding rock. The pressure above the hydrostatic pressure around the conduit (Fig. 5b) was found to not decrease and did not correspond to the observed seismic activity in the area. The observed discrepancy in pressure behavior can be attributed to the difference in permeability between the magma chamber and the surrounding rock.

Table 1 list all other fluid and rock parameters used in the model and a systematic parameter variation to constrain the influence of these assumptions on the results is presented in the Supplementary.

**Table 1:** Fluid and rock parameters used in the numerical model.

Symbol	Quantity	Value	Unit
$\beta$	Pore compressibility	$1e^{-10}$	1/Pa
$c_p$	Rock heat capacity	1.02	J/k/$^\circ$C
$\lambda$	Thermal conductivity	0.6	W/m/$^\circ$C
$\rho _r$	Rock density	2700	kg/m$^{3}$
$\rho _f$	Fluid density	900	kg/m$^{3}$
$\mu$	friction coefficient	0.8	–

The model assumes an initial temperature distribution, as depicted in Fig. 5a, that features elevated temperatures in the estimated magma chamber and magma conduit extending to shallow depths. The temperature of the rocks surrounding the magma chamber exhibits a conductive thermal gradient with a constant geothermal gradient of $50\,^{\circ}\hbox {C}$ per kilometer, which is a common feature in low permeability rocks outside volcanic regions. The steady state of the temperature distribution was calculated prior to the simulation. However, it is important to acknowledge that the system being modeled most likely also experiences hydrothermal convection driven by the magma chamber. Elevated temperatures at depths cause water rise transporting heat towards the surface, while lower temperatures occur in regions of downflow. This process is evidenced by the hot springs found on the edges of the Aso caldera, which indicate the occurrence of hydrothermal convection in the area.

The calculation of the steady state is based on the assumption of pure conduction, which does not account for hydrothermal convection. However, besides the necessary computational efforts, the shape of a hydrothermal convection cell is strongly dependent on subsurface heterogeneity (e.g. Kühn et al.). Non-seismically active faults and structures are not known with sufficient accuracy to include them into the model. The simplified model used in this study is sufficient for understanding the general seismic and heat behavior of the system. It provides a qualitative match of the spatiotemporal evolution of seismicity and predicts the temperature changes in the overlying geothermal system. However, it is important to keep in mind that the predictions derived from this model may not be highly accurate due to the simplifications made.

For heat transport, the viscous magma chamber from the surface to a depth of 10 km ranges from 140 to 1000 $^\circ$C, the temperature at the top boundary is set to 30 $^\circ$C in general but it is set to 140 $^\circ$C in the vicinity of the Nakedake volcano crater, as reported by Sudo et al.^83^ and Terada et al.^84^. Zero thermal flux boundary conditions are assumed for the left, right and bottom boundaries. The steady state background model in combination with the zero flux boundary condition results in a constant temperature at the lower boundary.

I assume an initial 40 MPa fluid overpressure pressure within the chamber and maintain the pressure until the October 8th, 2016 the volcanic eruption. As pressure builds up within the chamber over time, the duration of the simulation has been shortened by assuming an initial pressure instead of simulating the long-lasting pressure build-up. The chosen value is a reasonable assumption for magmatic chambers prior to an eruption^85^. The imposed pressure temperature field diffuses to a steady state before starting the simulation. At time = 0 (corresponding to 15 April 2016) a source term $Q_0 = 10^{-9}/{\text{s}}$ is imposed on the activated faults and in the magma chamber to simulate the degassing process of an active volcanic systems. We assume $Q_0$ because laboratory experiments are lacking for constraining S at relevant stresses and slip rates. Each of the chosen parameter values is a reasonably good choice based on the available data. However, the presented choices are non-unique as possibly several combinations of parameters exist providing similar results as the problem is underdetermined. The most loosely constrained parameter, at first glance, is the parameter $\alpha$ , which controls both the rate of ${\text {CO}}_2$ generation and post-seismic healing of the permeable network. The parameter itself reflects the substantial amount of fluid in the reservoir. Based on the observed earthquake data and the chosen set of parameters, alpha is varied to achieve the best fit between the spatio-temporal evolution of the 2016 Kumamato earthquake sequence and the numerical simulation.

## Supplementary Information

Supplementary Information.

Supplementary Video 1.

Supplementary Video 2.

## Data Availability

The dataset presented in this study are available in the online repositories of the Japanese Meteorological Agency http://www.jma.go.jp/jma/index.html. The simulation videos of fluid pressure diffusion and heat transport are made available through the following Github link: https://gitfront.io/r/Thanushika/vG9q7DLnzocG/Japan_Model/.
