# Supplementary material for: Dynamics between earthquakes, volcanic eruptions, and geothermal energy exploitation in Japan
Source: Sci Rep. 2023 Mar 21;13:4625. doi: 10.1038/s41598-023-31627-3 (PMC10030564; doi:10.1038/s41598-023-31627-3)
Supplement: Supplementary file 1 — Supplementary Information. [file 41598_2023_31627_MOESM1_ESM.pdf]

Supplementary Information for ’Dynamics between earthquakes, volcanic eruptions, and geothermal energy exploitation in Japan’

Thanushika Gunatilake[1,*]

[1]Center for Hydrogeology and Geothermics (CHYN), University of Neuchâtel, Neuchâtel, 2000, Switzerland
[*]Now at: ETH Zürich, Switzerland, Email: thanushika.gunatilake@sed.ethz.ch

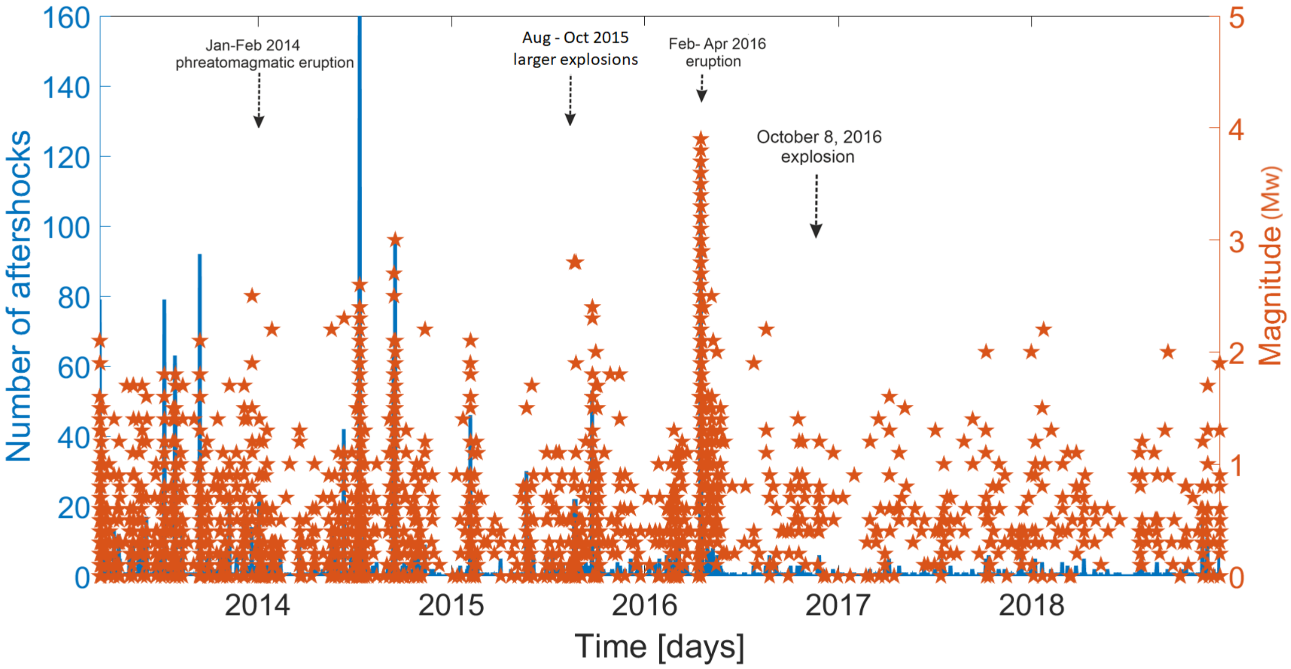

Supplementary Figure 1. Volcanic activity and seismicity. Earthquakes in the vicinity of the Aso caldera from 2013 to 2019 with their magnitude (Mw) compared to the volcanic activity of Mount Aso.

Figure 1 shows a time history of volcanic activity and seismic events from 2013 to 2019. This phenomenological comparison supports the well-known statement that volcanoes are responding to earthquakes and that they are always in interplay with each other. This volcano is known for pyroclastic eruptions, including ash emissions, strombolian eruptions, and phreatomagmatic activity[1,2]. The January and February 2014 preatomagmatic eruptions are accompanied by a number of earthquakes, with decreasing frequency in both number and magnitude of earthquakes observed. This might be explained by a pressure release through the eruptions. Similarly, in August to October 2015 larger volcanic explosions are associated with increased magnitudes and an increased frequency of earthquakes indicating the presence of high pressure fluids in the system. The highest magnitudes and the largest numbers of earthquakes are observed during February to April 2016 which coincide with volcanic eruptions. The seismic activity decreases remarkably after the large volcanic explosion on October 8, 2016, possibly due to the pressure release in the volcanic system. There are increases in seismic activity in June to August 2014, which are not related to volcanic activity at the surface. However, those can possibly be explained with subsurface dynamics of the volcano and fluid pressure increases in structures not connected to the surface. There are continuous observations at the surface measuring increased degassing of CO_2 and SO_2 during the whole period[3].

The irregular geometry shown in Figure 2a can be approximated by a finite difference domain with a regular grid. This approximation allows for the use of efficient numerical methods, such as implicit finite differences, to solve the equations on the discretized domain. The accuracy of the approximation will depend on the size and distribution of the nodes in the regular grid, as well as the representation of the boundaries of the irregular geometry. Using finite difference domains with regular

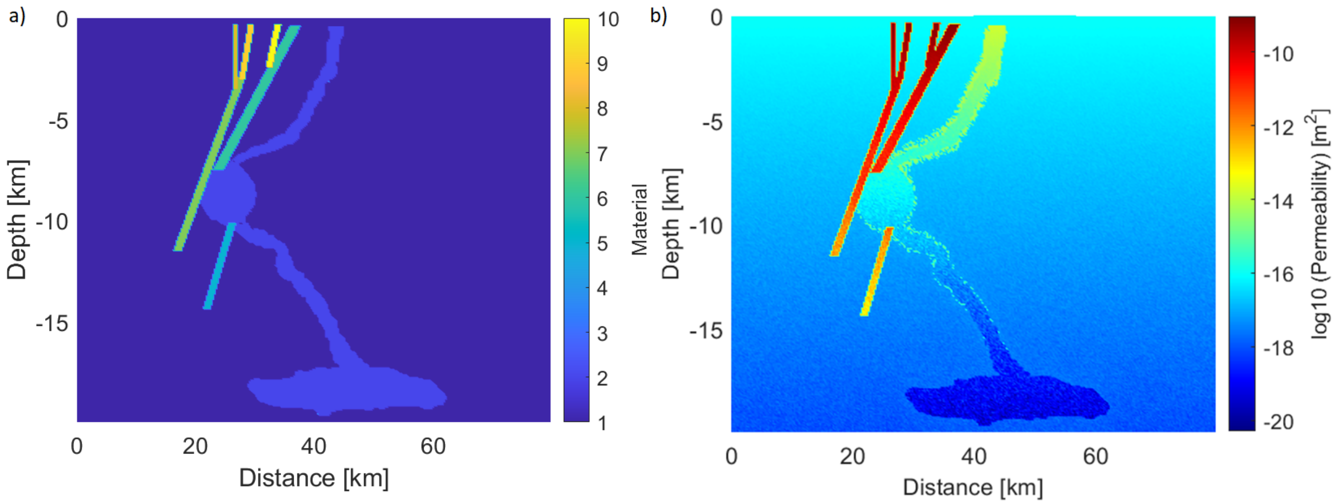

Supplementary Figure 2. Material matrix for 2D numerical simulation and initial Permeability conditions.

grids to represent irregular geometries can introduce uncertainties in the simulation results due to the approximation of the irregular geometry. Especially the boundary representation: The representation of the boundaries of the irregular geometry can introduce inaccuracies in the simulation results. For example, if the boundaries are represented as straight lines, then the representation of curved or angled boundaries will be less accurate. Despite these uncertainties, finite difference domains with regular grids are widely used to represent irregular geometries due to their computational efficiency and simplicity.

The initial conditions of the permeability distribution are depicted in Figure 2b. The permeability (k) is calculated based on the normal stress, leading to a heterogenous permeability with accelerated flow upwards and inhibited flow below the magma chamber, as demonstrated in[4]. The depth-dependence of permeability, as seen in crystalline rock in tectonically active areas, is taken into consideration in the model by linking fluid flow and seismicity.

To better understand the impact of different parameters, a sensitivity analysis can be performed. This type of analysis involves varying one parameter at a time, while holding all other parameters constant, and observing the resulting changes in the behavior of the system. This allows us to identify which parameters have the greatest impact on the system and to what extent. Figure 3 shows that parameter variations can greatly impact the spatiotemporal evolution of seismic events. Fluid and rock porosity, host rock permeability, and fault permeability all play crucial roles in determining the spatial and temporal characteristics of seismic events. Additionally, slight changes in the α value, which reflects the healing/sealing process of the permeability and controls the internal generation of the source over time, have a significant impact on the evolution of these events.

In hydrothermal convection, fluid properties such as density, viscosity, and compressibility are temperature and pressure-dependent and play a crucial role in controlling the thermo-hydraulic structure of the system. This study especially relies on a single-phase fluid model to represent the presence of both supercritical CO2 and water. A sensitivity analysis is conducted to evaluate the effect of these assumptions on the accuracy of the simulation results and allows us to assess the robustness of the presented model.

Figure 4 shows the sensitivity analysis of the properties, such as fluid and rock density, compressibility, and viscosity. It appears that the changes in the initial values of the parameters had only a minor effect on the numerical seismic events. This is a strong indication that the model is robust and that the chosen parameter values are reasonable. However, it is evident that among the parameters studied, variations in density had the most pronounced effect on the simulation results. This highlights the importance of accurately modeling the density of supercritical CO2 and water in hydrothermal convection systems as a two-phase fluid model in further studies.

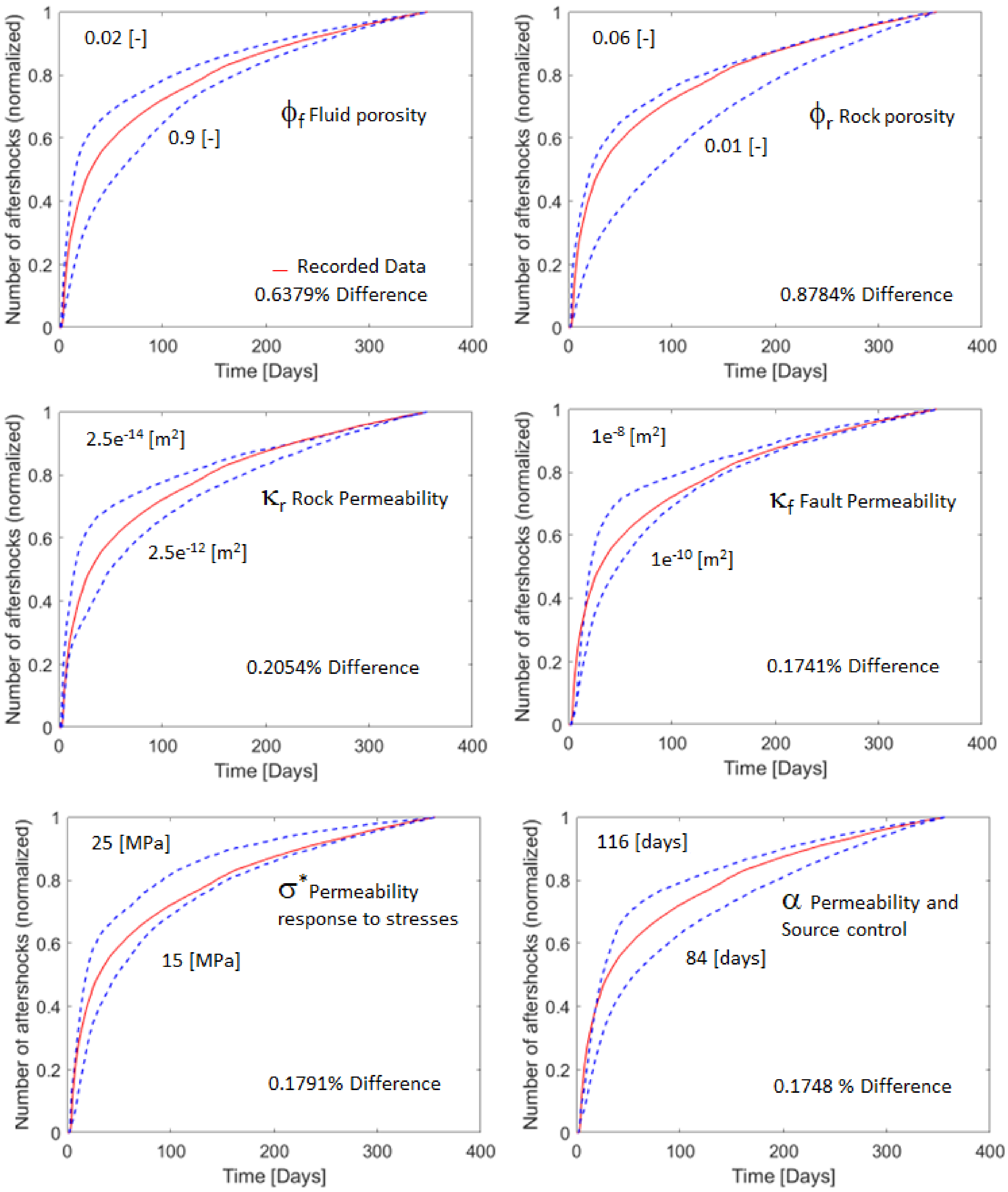

Supplementary Figure 3. Parameter variations indicating that changes in fluid and rock porosity, host rock and fault permeability, and especially slight changes in α value strongly influence the spatiotemporal evolution of seismic events.

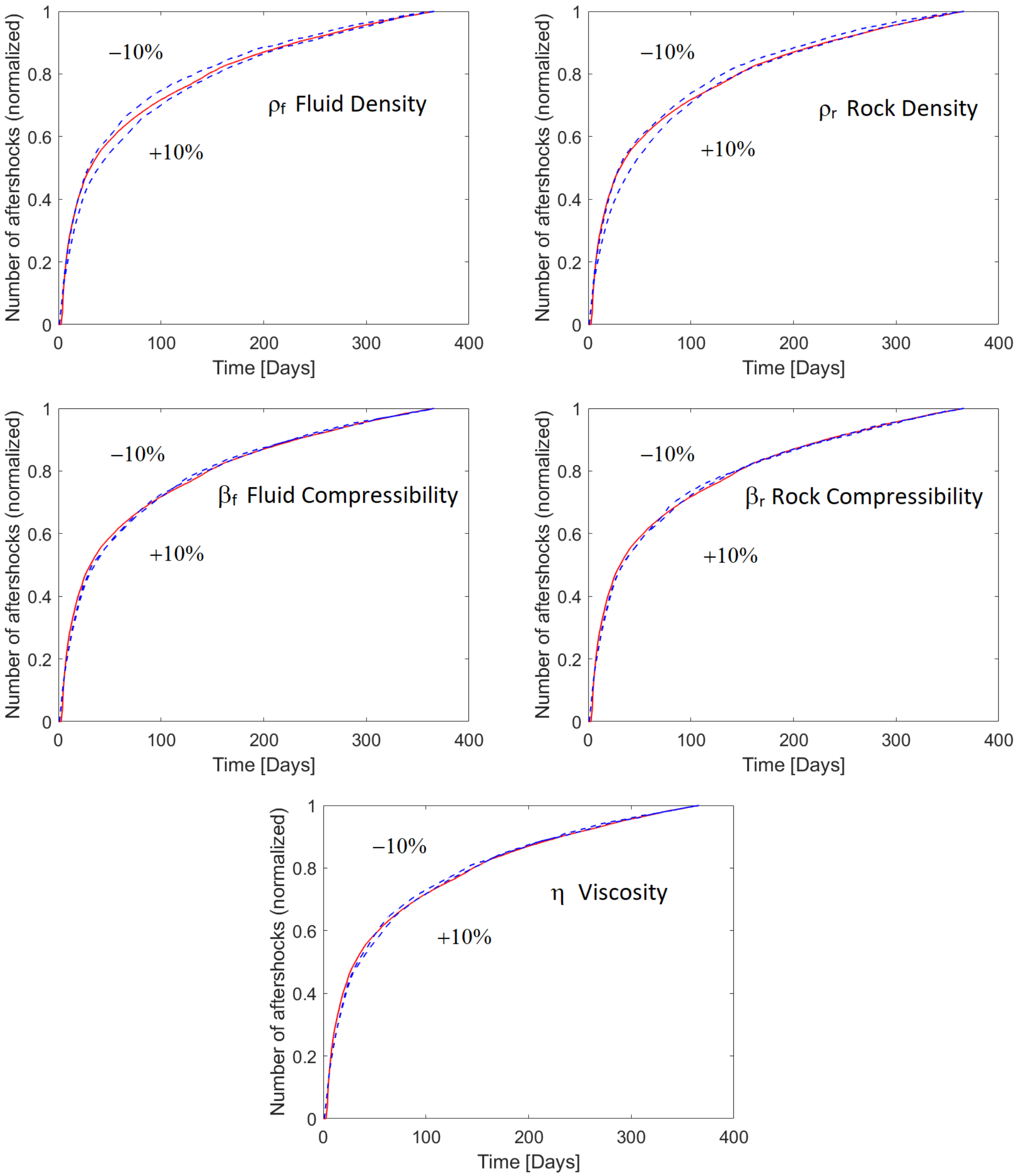

Supplementary Figure 4. Parameter variations indicating that changes in fluid and rock density, compressibility, and viscosity by $\pm$ 10 % of the initial value described in the method section, have little influence on the spatiotemporal evolution of seismic events, by comparing the numerical seismic events and the recorded seismic data.

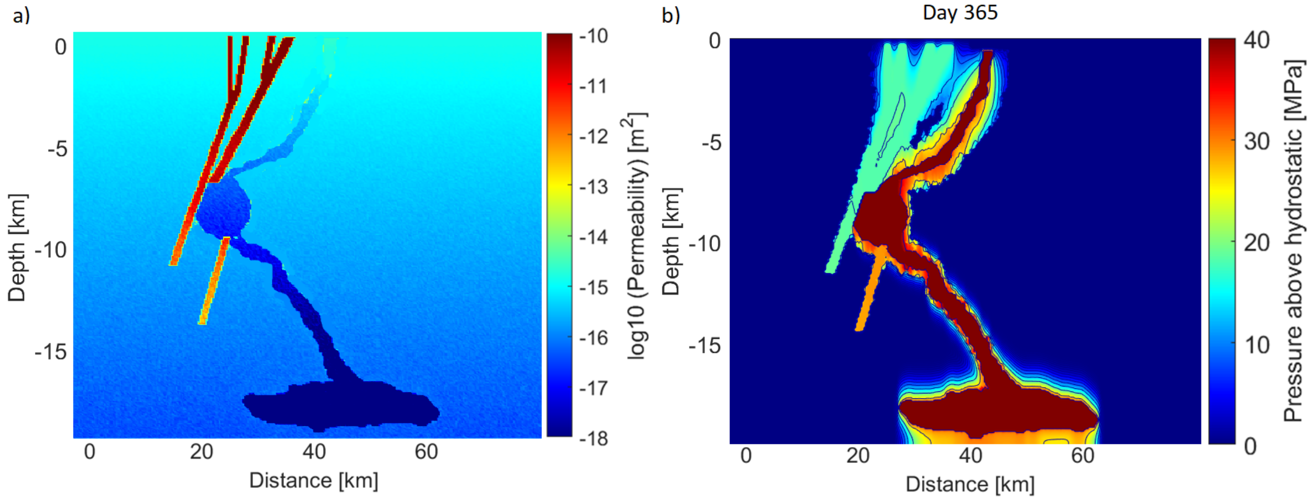

Supplementary Figure 5. Permeability of the magma chamber is lower than that of the surrounding rock. The pressure above the hydrostatic pressure around the conduit does not exhibit a decrease and does not align with the observed seismic activity in the study area.

References

1. Ono, K., Watanabe, K., Hoshizumi, H. & ichiro Ikebe, S. Ash eruption of the Naka-dake crater, Aso volcano, southwestern Japan. *J. Volcanol. Geotherm. Res.* **66**, 137–148, DOI: 10.1016/0377-0273(94)00061-K (1995).

2. Lin, A. *The 2016 Mw 7.1 Kumamoto Earthquake: A Photographic Atlas of Coseismic Surface Ruptures Related to the Aso Volcano, Japan* (Springer, 2017).

3. Morita, M., Mori, T., Yokoo, A., Ohkura, T. & Morita, Y. Continuous monitoring of soil co 2 flux at aso volcano, japan: the influence of environmental parameters on diffuse degassing. *Earth, Planets Space* **71**, 1–16 (2019).

4. Hamidi, S., Heinze, T., Galvan, B. & Miller, S. A. Numerical study of asymmetric vertical fluid intrusion in deep reservoirs: Effects of stress, temperature and salinity. *Tectonophysics* **750**, 280–288 (2019).

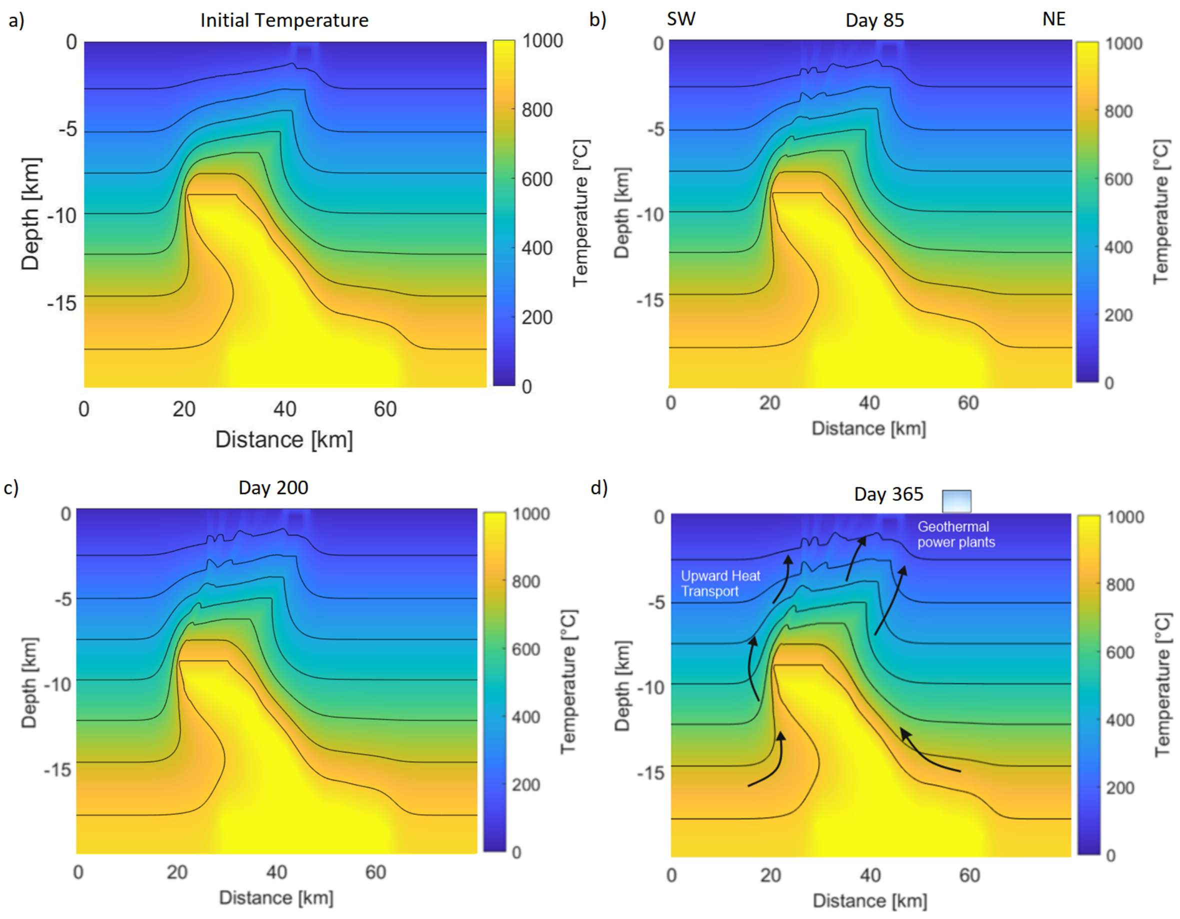

Supplementary Figure 6. Advection-Diffusion of temperature for different time steps. a) Initial temperature distribution after 200 years of thermal conduction. b-c) The heat migrate from the magma chamber to the faults and upwards within the conduit. d) Temperature distribution at day 365 and location of geothermal power plants illustrating the upward migration of the heat.

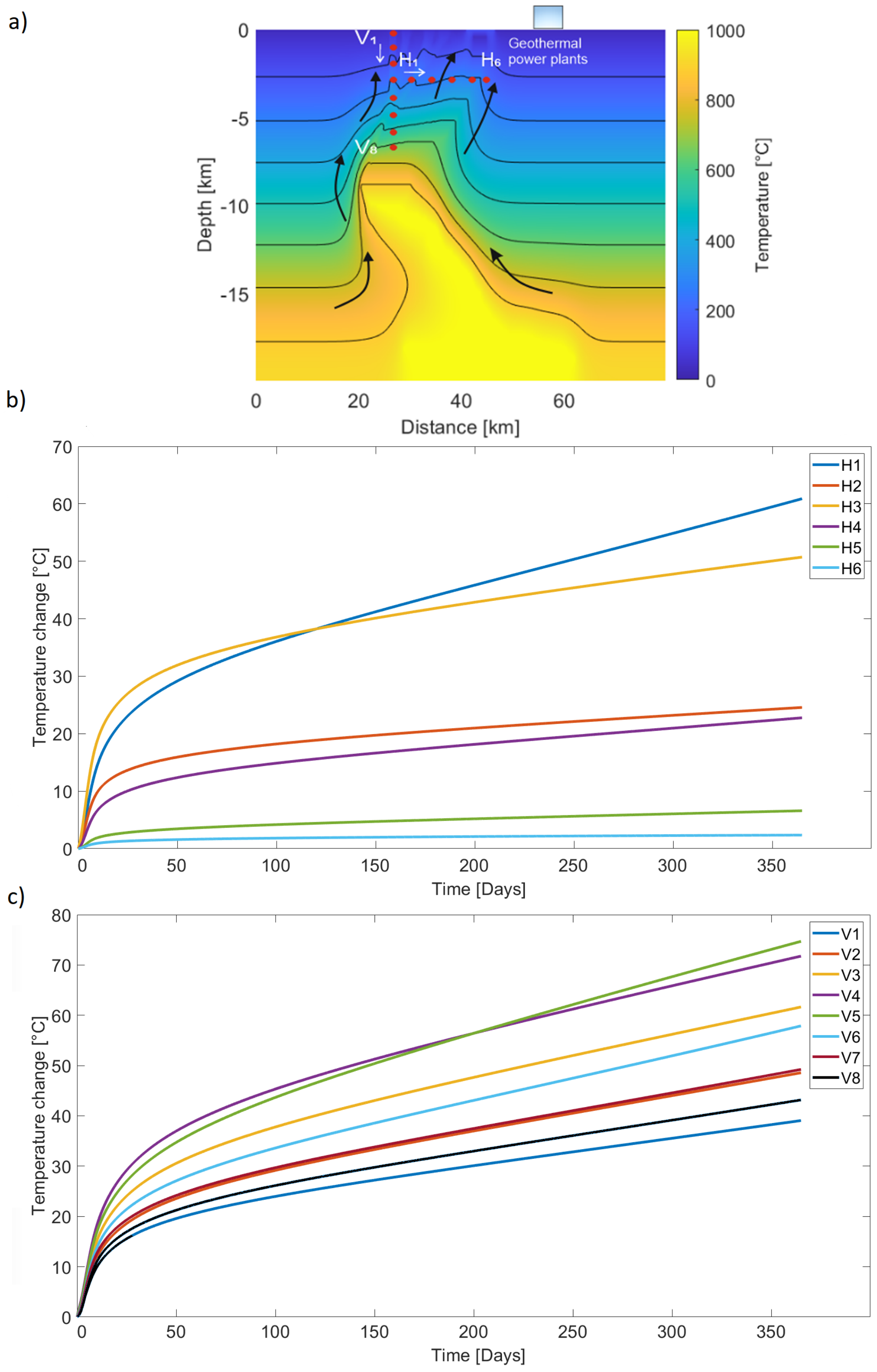

Supplementary Figure 7. Temperature profiles. a) Temperature distribution at 365 simulation day. The red dots indicate the location of the vertical and horizontal profiles. b) Horizontally aligned temperature profiles (H1-H6) with a vast difference in temperature distribution. c) Nine temperature distribution profiles show the temperature increase from top (V1) to bottom (V8). The smoother gradients with larger temperature increases in the surrounding rock cause higher temperature changes.
